# TAT as a new marker and its use for noninvasive chemical biopsy in NASH diagnosis

**DOI:** 10.1186/s10020-024-00992-8

**Published:** 2024-11-26

**Authors:** Sihyang Jo, Jin-Mo Kim, Minshu Li, Han Sun Kim, Yong Jin An, Sunghyouk Park

**Affiliations:** 1 Natural Products Research Institute, College of Pharmacy, Seoul National University, Gwanak- Ro 1, Gwanak-gu, Seoul, 08826 Republic of Korea; 2 Department of Biochemistry, College of Medicine, Dongguk University, Gyeongju, 38066 Republic of Korea

**Keywords:** Nonalcoholic steatohepatitis, Biomarker, Noninvasive, Chemical biopsy, Liquid biopsy, Stable isotopes

## Abstract

**Background:**

Early diagnosis of Nonalcoholic steatohepatitis (NASH) is crucial to prevent its progression to hepatocellular carcinoma, but its gold standard diagnosis still requires invasive biopsy. Here, a new marker-based noninvasive chemical biopsy approach is introduced that uses urine-secreted tyrosine metabolites.

**Methods:**

We first identified NASH-specific decrease in TAT expression, the first enzyme in the tyrosine degradation pathway (TDP), by employing exometabolome-transcriptome correlations, single-cell RNA -seq, and tissue staining on human NASH patient samples. A selective extrahepatic monitoring of the TAT activity was established by the chemical biopsy exploiting the enzyme’s metabolic conversion of D_2_-tyrosine into D_2_-4HPP. The approach was applied to a NASH mouse model using the methionine-choline deficient diet, where urine D_2_-4HPP level was measured with a specific LC-MS detection, following oral administration of D_2_-tyrosine.

**Results:**

The noninvasive urine chemical biopsy approach could effectively differentiate NASH from normal mice (normal = 14, NASH = 15, *p* = 0.0054), correlated with the NASH pathology and TAT level decrease observed with immunostaining on the liver tissue. In addition, we showed that the diagnostic differentiation could be enhanced by measuring the downstream metabolites of TDP. The specificity of the TAT and the related TDP enzymes in NASH were also addressed in other settings employing high fat high fructose mouse NASH model and human obesity vs. NASH cohort.

**Conclusions:**

Overall, we propose TAT and TDP as pathology-relevant markers for NASH and present the urine chemical biopsy as a noninvasive modality to evaluate the NASH-specific changes in urine that may help the NASH diagnosis.

**Supplementary Information:**

The online version contains supplementary material available at 10.1186/s10020-024-00992-8.

## Background

Nonalcoholic steatohepatitis (NASH), also known as metabolic dysfunction-associated steatohepatitis (MASH), is a type of metabolic dysfunction-associated steatotic liver disease (MASLD). NASH is the second most common cause of liver transplantation in the United States, highlighting its significant clinical impact (Noureddin et al. 2018), (Kabbany et al. 2017). It is associated with hepatocellular injury, inflammation, and fibrosis that can progress to cirrhosis. In severe cases, it significantly increases the risk of developing hepatocellular carcinoma (Ascha et al. 2010), (Brown and Kleiner 2016). Therefore, it is important to diagnose NASH as quickly and accurately as possible, allowing resources to be directed to those most in need and helping to prevent adverse outcomes associated with the disease (Cotter and Rinella 2020). 

A liver biopsy is considered the gold standard for diagnosing NASH. This involves removing of a small amount of liver tissue with a needle, which is then examined under a microscope to identify the presence of fat, inflammation, and fibrosis. However, this method has its limitations, including cost, the potential for internal bleeding and injury to other organs, as well as risk of infection (Chalasani et al. 2018). In addition, can misdiagnose NASH in up to 25% of samples (European Association for the Study of the Liver (EASL); European Association for the Study of Diabetes (EASD); European Association for the Study of Obesity (EASO) 2016). Therefore, noninvasive testing (NIT), such as physical examination, liquid biopsy, and imaging procedures, have been proposed to identify NASH without liver biopsy (Mauro et al. 2021). Liquid biopsy has been increasingly recognized as a noninvasive, quick, and cost-effective method for identifying biomarkers in biofluids such as blood and urine (Zhou et al. 2016), (Mattox et al. 2019). And imaging techniques such as ultrasonography, computed tomography (CT), and magnetic resonance imaging (MRI) have been employed for directly detecting liver fat and assessing liver disease. However, it should be noted that these NIT lack the liver tissue specificity required for NASH identification and do not reliably reflect the histologic spectrum (Chalasani et al. 2018), (Castera et al. 2019). Therefore, as no single NIT has yet proven accurate enough to supplant a liver biopsy, it remains the gold standard for diagnosing NASH, underscoring the need for the development of NASH diagnostic modalities (Wieckowska et al. 2007), (Zhou et al. 2019). 

In this regard, the concept of ‘chemical biopsy’, a type of liquid biopsy, has gained attention, involving the detection of specific tissue-derived metabolites (biomarkers) in biofluids such as urine and blood (Wagner 1970), (Landau 1991). It has been used in previous studies to observe and evaluate the specific activities of key hepatic metabolic intermediates such as acetyl-CoA, TCA intermediates, and UDP-glucose in urine and blood by oral administration of drugs or tracers to assess liver function (Hellerstein et al. 1987), (Hellerstein et al. 1991), (Jones et al. 2001). Recently, the use of stable isotopes that can track and evaluate cancer-specific metabolism in chemical biopsy for early diagnosis of liver cancer has been proposed (Cha et al. 2021), (Oh et al. 2023), but a further understanding of disease- and tissue-specific metabolism is still needed.

Compared to healthy individuals, NASH patients exhibit significant disruptions in several key amino acid metabolic pathways, alongside lipid metabolism (Masoodi et al. 2021), (Niu et al. 2022). Particularly, indications from amino acid metabolism suggest that levels of tyrosine-containing aromatic amino acid (AAAs) and branched chain amino acid (BCAAs) are increased in NASH patients (Cheng et al. 2015), (Mello et al. 2021). Proposed reasons for the elevated levels of amino acid (AA) in the circulation in NASH include increased rates of whole-body protein turnover and impaired AA catabolism occurring in the later stages of steatosis progression to NASH (Newgard 2012), as well as overall impaired hepatic metabolism of AAA due to intrahepatic lipid accumulation (Cheng et al. 2015), (Dejong et al. 2007). It is also observed that these serum tyrosine levels are associated with insulin resistance, steatosis, and the fibrotic phase of NASH (Cheng et al. 2015), (Kawanaka et al. 2015), (Gaggini et al. 2018). Consequently, not only in serum but also in urinary metabolomics, differences in metabolites are observed compared to normal (Dong et al. 2017), with some researchers suggesting that tyrosine metabolism is the most affected of these metabolic changes (Jin et al. 2016). However, despite clear differences in amino acid metabolic phenotypes in previous studies on NASH diagnosis, there is a dearth of molecular evidence and its application for diagnosis.

Tyrosine aminotransferase (TAT) acts as the rate-limiting enzyme in the tyrosine degradation pathway in liver cells, where it plays a critical role by converting tyrosine into 4-hydroxyphenylpyruvate (4HPP) (Dickson et al. 1981). Deficiency of this enzyme is associated with Tyrosinemia type II, a hereditary metabolic disorder characterized by elevated blood tyrosine levels (Natt et al. 1992). Additionally, recent functional analyses have demonstrated that TAT possesses pro-apoptotic effects, and its suppression may contribute to the promotion of liver tumorigenesis (Fu et al. 2010). 

Here, we identified the candidate noninvasive chemical biopsy targets for NASH-specific diagnosis in the liver through exometabolome-transcriptome correlation studies, developed a stable isotope-based urine chemical biopsy approach, and evaluated its relevance in detecting asymptomatic and early molecular pathological manifestations of NASH.

## Materials and methods

### Western blot analysis

Cells and Tissues were lysed with RIPA buffer (50 mM Tris-HCl, pH 7.4; 1% Nonidet P-40; 0.25% sodium deoxycholate; 150 mM NaCl; 1 mM Na_3_VO_4_) that included a cocktail of protease inhibitors (Cat# 271306) and phosphatase inhibitors (Cat# ab201112, Abcam, Toronto, ON, Canada). Western blot analysis was performed following a standard protocol. The antibodies below were used to detect each specific protein, anti-TAT (sc-376292), anti-β-actin (sc-47778, Santa Cruz, Dallas, TX, USA), goat anti-mouse IgG-HRP (sc-2005, Santa Cruz, Dallas, TX, USA) antibodies.

### Immunohistochemistry

A human-tissue microarray (TMA) for liver normal and steatohepatitis with no history of alcohol use (Cat# TMA.NASH, Lot# 2110289, XENOTECH, Kansas City, KS, US) was examined for TAT expression. Pathology and alcohol consumption information was provided by the vendor. The tissues were stained according to standard immunohistochemistry protocol using an antibody for TAT (sc-376292 Santa Cruz, Dallas, TX, USA) and hematoxylin.

### AST/ALT measurement

After anesthesia, whole blood was collected from the mouse heart, coagulated for 30 min at room temperature, and then centrifuged (1,000 g, 10 min, 4℃). The upper layer was carefully transferred to a new tube and stored at -20 °C until experiments. To detect serum AST and ALT activities, serum was applied on slides (GOT/AST-P III, GPT/ALT-P III, Fuji Dri-Chem; Fujifilm, Kanagawa, Japan) and analyzed using a biochemical blood analyzer (DRI-CHEM 3500s, FUJIFILM, Japan) at the Animal Center for Pharmaceutical Research of Seoul National University. The procedure was performed according to the manufacturer’s protocol.

### Cell preparation and stable isotope labeling

Primary hepatocytes were cultured in DMEM medium (Cat# LM 001–05, Welgene, Gyeongsan, Korea) supplemented with 10% FBS (Cat# S 001–07, Welgene) and 1% penicillin streptomycin (Cat# LS 203-01, Welgene) at 37℃ in a 5% CO_2_ humidified incubator. Cells were seeded in 6 well culture plate and incubated with 1 mM D_2_-tyrosine (RING-3,5-D_2_, 98%, Cat# DLM-449, Cambridge Isotope Laboratories, Inc., Andover, MA, USA) for overnight. The media containing the stable isotope is then removed, washed twice with PBS, replaced with fresh media, and incubated overnight. The media were collected and extracted for the detection of metabolites.

### Design of NASH animal model and stable isotope administration

All protocols involving the use of animals were approved by the Institutional Animal Care and Use Committees (IACUC) of Seoul National University, Seoul, Korea (SNU-200928-3-3), and were performed in accordance with the relevant guidelines. For the mouse liver NASH model, we used methionine choline deficient (MCD) diet-induced model. It is an autochthonous NASH model and was performed as reported previously (Kucera 2014). Briefly, male C57BL/6 mouse (Orient Bio, Gapyeong, South Korea) weighing 20 g were provided with MCD diet (Cat# A02082002BR, Research Diets, USA) for the 5 weeks. The control group received no MCD diet. There were 15 animals randomly grouped in each group, and one animal died in the control group during the experiment, leaving 14/15 animals per group. After 5 weeks the animals were fasted for 4 h, after which D_2_-tyrosine (200 mg/kg) was administered orally. The urine was collected for 3 h in the metabolic cage after the oral administration of D_2_-tyrosine.

### Aqueous and the organic metabolite sampling in mouse liver tissue

For analysis of metabolite level in liver tissue, two-phase extraction was employed as follows. Twentyfive milligrams of liver tissue was quickly cooled with liquid N_2_ and ground to a fine powder using a mortar and pestle. The powder was transferred to a tube, added 100 µL of methanol plus 50 µL of chloroform, ground with a hand homogenizer, and added 300 µL of methanol and 150 µL of chloroform. The sample was frozen with liquid N_2_ for 1 min, thawed at room temperature for 1 min, and mixed using a vortex mixer for 1 min. This process was repeated three times. Then, 200 µL of chloroform and 200 µL of distilled water were added, and the lysates were separated by centrifugation (18,000 g, 30 min, 4℃). After centrifugation, the aqueous layer and organic layer were carefully transferred into new 1.5 mL tubes. The water layer, the organic layer and the pellet were completely dried with a CentraVac (Vision, Gyeonggi-do, Korea). The aqueous layer and the organic layer were stored at − 20 °C until further analysis. The protein pellet was used for protein normalization.

### NMR measurement

The lipid-soluble layer was dissolved in 500 µL pure *d*-chloroform. The samples were transferred to a 5 mm NMR sample tube. The NMR spectra were acquired using an 800 MHz Bruker Avance spectrometer equipped with a cryogenic triple-resonance probe (Bruker, Billerica, MA, USA) at College of Pharmacy, Seoul National University, Seoul, Korea. The 2D-HSQC spectra were obtained using the Bruker pulse sequence hsqcetgpsisp2.2. The spectral widths were set to 16 ppm (H) and 80 ppm (C), with 2048 (T2) × 128 (T1) complex points and 8 number of scans per one indirect point.

### Extraction and MS sample preparation

Methanol (400 µL) and chloroform (200 µL) were added to media (or urine) (200 µL). Then, the following procedure was repeated three times; snap freeze in liquid N_2_ (1 min), thaw on ice (3 min), and vortex for 30 s. Additional 200 µL of chloroform were added and the sample was vortexed for 1 min. Then the sample was centrifuged at 18,000 g for 30 min at 4℃. After centrifugation, the aqueous layer was carefully transferred into a new microcentrifuge tube. The samples were dried using CentraVac (Vision, Gyeonggi-do, Korea). The dried samples were stored − 20℃ until measurements. The dried samples were dissolved in 50 µL of water:acetonitrile (ACN) (1:1 v/v) and centrifuged at 18,000 g at 4 °C for 20 min and a total of 3 µL was into the LC-MS spectrometer. Metabolites were separated using an ACQUITY UPLC BEH Amide Column (130Å, 1.7 μm, 2.1 mm X 100 mm) at 40 °C connected to a Triple Quad LC/MS Mass Spectrometer (1290 Infinity, Agilent). The data was analyzed with MassHunter Software (Agilent, United States). Metabolites were detected in negative polarity. The mobile phases were 10 mM ammonium acetate in 100% water (A) and 10 mM ammonium acetate in water: ACN (1:4) (B) with the gradient as follows: 0% A at 0 min, 0% A at 2 min, 40% A at 7.5 min, 40% A at 16 min, 0% A from 16.5 to 20.1 min with a 0.2 mL/min flow rate. For urine, measurements in MS were performed by shuffling the order of the samples. The D_2_-tyrosine and D_2_-4HPP level was normalized with urinary creatinine of each paired samples.

### Single-cell RNA-seq analysis

The scRNA-seq data obtained from Su and colleagues (Su et al. 2021) were subjected to reanalysis using the Seurat pipeline. In order to ensure data quality, cells exhibiting fewer than 200 RNA features and a high mitochondrial fraction (> 10%) were excluded. Subsequently, the data underwent normalization by dividing the total unique molecular identifier (UMI) counts of each cell. The Seurat guideline (http://satijalab.org/seurat/) was followed for clustering, normalization, and batch correction procedures. Hepatocytes were specifically filtered, and subsequent steps involved neighbor identification and clustering. The resulting data were visualized in two dimensions through Uniform Manifold Approximation and Projection (UMAP) plots. Dimensional plots were presented according to weeks, and feature plots were utilized to display *TAT*. For additional scRNA analysis, data from other studies (Su et al. 2021), (Guilliams et al. 2022) available on different scRNA-seq web database sites were used; Liver Cell Atlas (https://www.livercellatlas.org/index.php), Single Cell PORTAL (https://singlecell.broadinstitute.org/single_cell), and Azimuth (https://azimuth.hubmapconsortium.org).

### Bioinformatics analysis

MetaboAnalyst 5.0 was used for enrichment analysis and overrepresentation analysis and was applied to the GEO dataset. Only significantly different genes (*p*-value < 0.05) determined by unpaired student’s *t*-test were used for diagnostic candidate selection.

### Statistical analysis

Results were measured in at least three biologically independent samples unless otherwise indicated in the figure. The graphs were presented as mean values and standard deviations using the GraphPad Prism v9.3.0 program. The statistical significance between groups was calculated using an unpaired Student’s *t*-test. In the graphs, an asterisk (“*”) indicates a statistically significant *p*-value. ns. not significant, * *p* < 0.05, ** *p* < 0.01, *** *p* < 0.001, **** *p* < 0.0001.

## Results

### Screening of liver tissue metabolism-specific biomarkers for the diagnosis of NASH

**Fig. 1:**
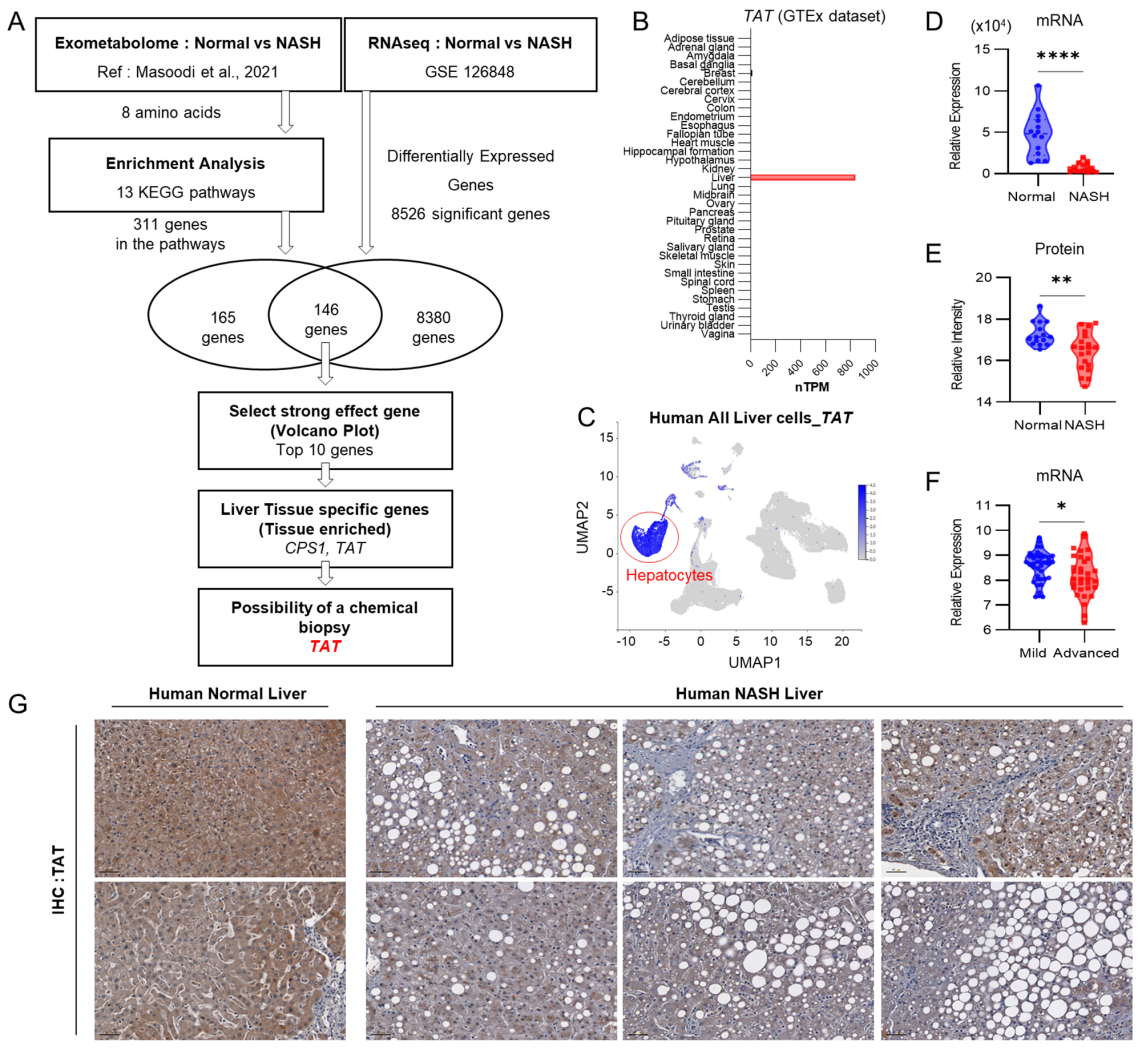
TAT is a biomarker for NASH. (**A**) Scheme of the screening procedure for finding the chemical biopsy target in NASH. **(B)** Expression of *TAT* in human normal tissues. The RNA expression of the normal tissues was normalized using the internal normalization pipeline (Expression (nTPM) levels for 35 tissue types, created by combining the HPA and GTEx transcriptomics datasets). **(C)** Single-cell RNA-seq data for expression of *TAT* in human all liver cells. Hepatocytes are indicated with red circles. **(D-G)** Expression of *TAT* in NASH. **(D)** mRNA levels of *TAT* in normal and NASH patients from GSE126848. **(E)** Protein expression of TAT in normal and NASH patients from liverproteome. (**F**) mRNA levels of *TAT* in the progression of NASH patients from GSE31803. (**G**) IHC staining of TAT in human tissue array section. Magnification 20×. Scale bar 100 μm. Statistical analysis was performed in comparison with the normal group by unpaired Student’s *t*-tests. ns. not significant, * *p* < 0.05, ** *p* < 0.01, *** *p* < 0.001, **** *p* < 0.0001. Data in panels (**B**) was acquired from The Human Protein Atlas (HPA) database. Data in panels (**C**) was acquired from Liver Cell Atlas

To discover new biomarkers for the diagnosis of NASH by noninvasive chemical biopsy, an exometabolome-transcriptome correlation analysis was performed. We designed this approach to find a NASH-related gene that can modulate extracellular metabolite, which, in turn, can be exploited for noninvasive detection. For the exometabolome-centric search, literature review was performed to identify differences in extracellular metabolites between normal and NASH groups. It was found that the blood of NASH patients had higher levels of eight amino acids, including isoleucine, leucine, valine, phenylalanine, tyrosine, arginine, lysine, and threonine, than the normal group (Masoodi et al. 2021). Enrichment analysis using KEGG pathways for these metabolic markers gave 13 metabolic pathways including ‘phenylalanine, tyrosine and tryptophan biosynthesis’, encompassing 311 genes (Figure S1A). Then, a transcriptome-centric search was performed where RNA-seq data from Human GEO database (GSE126848) was analyzed for differential expression between NASH and normal patients (criterion: *p* < 0.05; Table S1), giving 8526 genes. Intersecting the two results gave 146 genes, which were further narrowed down to 10 by log_2_ fold changes in the volcano plot (Figure S1B and Table S2). Final target selection was performed manually to assure the liver-specific protein expression of these 10 genes on Human Protein Atlas, which identified *CPS1* (carbamoyl-phosphate synthase 1) and *TAT* (tyrosine aminotransferase) as possible candidates (Fig. 1A, B and Table S3). For these two, the suitability for noninvasive chemical biopsy was assessed based on the nature of their enzymatic reactions (Table S4). For CPS1, the enzyme uses ATP, bicarbonate, and ammonium ion as substrates to produce ADP and carbamoyl phosphate. However, these products are poorly cell-permeable and unstable. The other candidate, TAT, uses tyrosine as a substrate to generate 4-hydroxyphenylpyruvate (4HPP), which is known to be cell permeable, non-toxic, and detectable in urine. Therefore, for the development of noninvasive chemical biopsy, the reaction of TAT was found to be more suitable than that of CPS1. To enhance the reliability of TAT as a liver tissue metabolism-specific biomarker for NASH diagnosis, we further investigated its feasibility using various databases. Single-cell RNA-seq data (Guilliams et al. 2022), (Fabre et al. 2023) analysis revealed that *TAT* exhibits hepatocyte-specific expression, which is responsible for liver function, indicating its potential as a biomarker for this fundamental liver cell type (Fig. 1C and Figures S1C, D). Consistent with this finding, the Azimuth website identifies *TAT* as one of the most significant gene factors distinguishing hepatocytes from other liver cell populations (Figure S1D). The relevance of TAT expression in NASH was further validated by its lower levels in independent mRNA and protein (Niu et al. 2022) datasets (Fig. 1D, E). Furthermore, the GSE31803 data set indicated lower *TAT* levels in advanced NASH stages compared to milder stages (Fig. 1F). These findings were also independently confirmed using human tissue microarrays (TMA) for normal liver and steatohepatitis, demonstrating a clear decrease in TAT expression with progressive NASH (Fig. 1G). These results showed that hepatic TAT levels decrease with NASH progression. It indicated the utility of TAT as a novel biomarker and suggested its usefulness in noninvasive chemical biopsy for NASH diagnosis.

### Design of a chemical biopsy strategy based on TAT enzyme activity

**Fig. 2:**
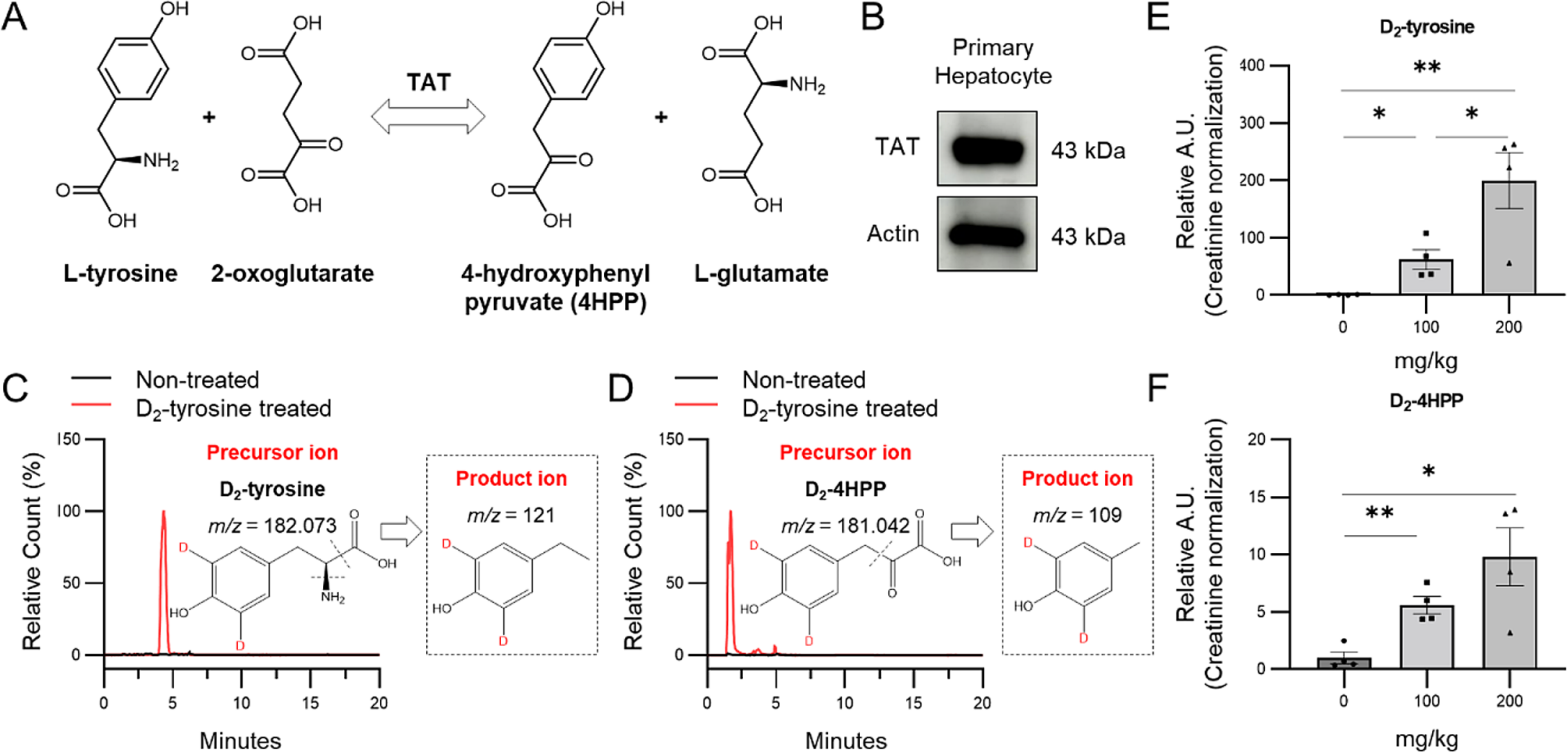
Design of chemical biopsy for the measurement of TAT activity. (**A**) Tyrosine degradation pathways for 4HPP formation by TAT. (**B**) Confirmation of TAT expression in primary hepatocytes using western blot. (**C-D**) Design of the MRM for the detection of D_2_-tyrosine and D_2_-4HPP. The red “D” indicates the positions of the deuterium labels. Chromatogram of D_2_-tyrosine (**C**) and D_2_-4HPP (**D**) measured in primary hepatocyte culture medium. The dotted inset shows the structure of the fragment ion containing deuterium. Chromatogram black line: medium without D_2_-tyrosine, red line: medium with D_2_-tyrosine. (**E-F**) Evaluation of TAT activity in response to changes in tyrosine concentration in normal mice measured by chemical biopsy (0, 100, 200 mg/kg, *n* = 4). Comparison of urinary D_2_-tyrosine (**E**) and D_2_-4HPP (**F**) levels between the three dose groups of D_2_-tyrosine feeding. Data were obtained from biologically independent mouse urine samples (*n* = 4 per group)

To exploit the TAT activity for noninvasive NASH diagnosis, an analytical scheme was designed based on the TAT-catalyzed reaction. The metabolism of tyrosine by TAT begins with transamination to produce 4HPP (Fig. 2A). Therefore, we reasoned that TAT activity could be assessed by measuring the newly formed 4HPP. As it is needed to track only the newly generated 4HPP by TAT in the liver, excluding the pre-existing 4HPP in vivo, D_2_-tyrosine was used. As deuterium is non-radioactive, this compound can be used without any radiation-safety regulation. In addition, specific LC-MS multiple reaction monitoring (MRM) schemes were designed for the detection of the substrate (D_2_-tyrosine) and product of TAT (D_2_-4HPP). For D_2_-tyrosine (C_9_H_9_D_2_NO_3_), a Q1 *m/z* value of 182.073 and a Q3 *m/z* value of 121 (C_8_H_6_D_2_O) were measured; for D_2_-4HPP (C_9_H_6_D_2_O_4_), a Q1 *m/z* value of 181.042 and a Q3 *m/z* value of 109 (C_7_H_6_D_2_O) were measured. To determine whether this MRM method, which selectively measures only fragments containing deuterium in the ring, could successfully exclude pre-existing tyrosine and 4HPP, the method was applied to primary hepatocytes were tested. First, the expression of TAT protein was confirmed in primary hepatocytes by Western blot, indicating that primary hepatocytes can be used for in vitro testing (Fig. 2B, S2). Then, the cells were incubated with D_2_-tyrosine for 16 h, followed by washing with PBS to eliminate any residual D_2_-tyrosine and subsequent replacement with fresh medium (refer to Methods for detailed protocol). Finally, the medium was analyzed to detect the presence of D_2_-4HPP, a metabolite generated by intracellular TAT, indicating enzymatic activity. The results showed that peaks of D_2_-tyrosine and D_2_-4HPP were detected and measurable by LC-MS only when treated with D_2_-tyrosine. Conversely, they were absent in the media of untreated cells (Fig. 2C, D). The absence of detectable deuterium isotope-labeled endogenous metabolites means that these D-labeled metabolites cannot be produced by other pathways and are formed only from D_2_-tyrosine metabolized by TAT. Then, the designed approach was further validated in vivo system to assess whether D_2_-4HPP, produced from D_2_-tyrosine by TAT, could be detected in urine, offering a potential noninvasive biomarker for liver TAT activity. When different doses of D_2_-tyrosine were orally administered to mice, both D_2_-tyrosine and D_2_-4HPP were successfully measured in the urine in a dose-dependent manner (Fig. 2E, F). Since liver TAT levels are decreased as NASH progresses our results provide a proof of concept for the assessment of NASH by noninvasive urine chemical biopsy of D_2_-4HPP.

### Chemical biopsy for noninvasive diagnosis of NASH

**Fig. 3:**
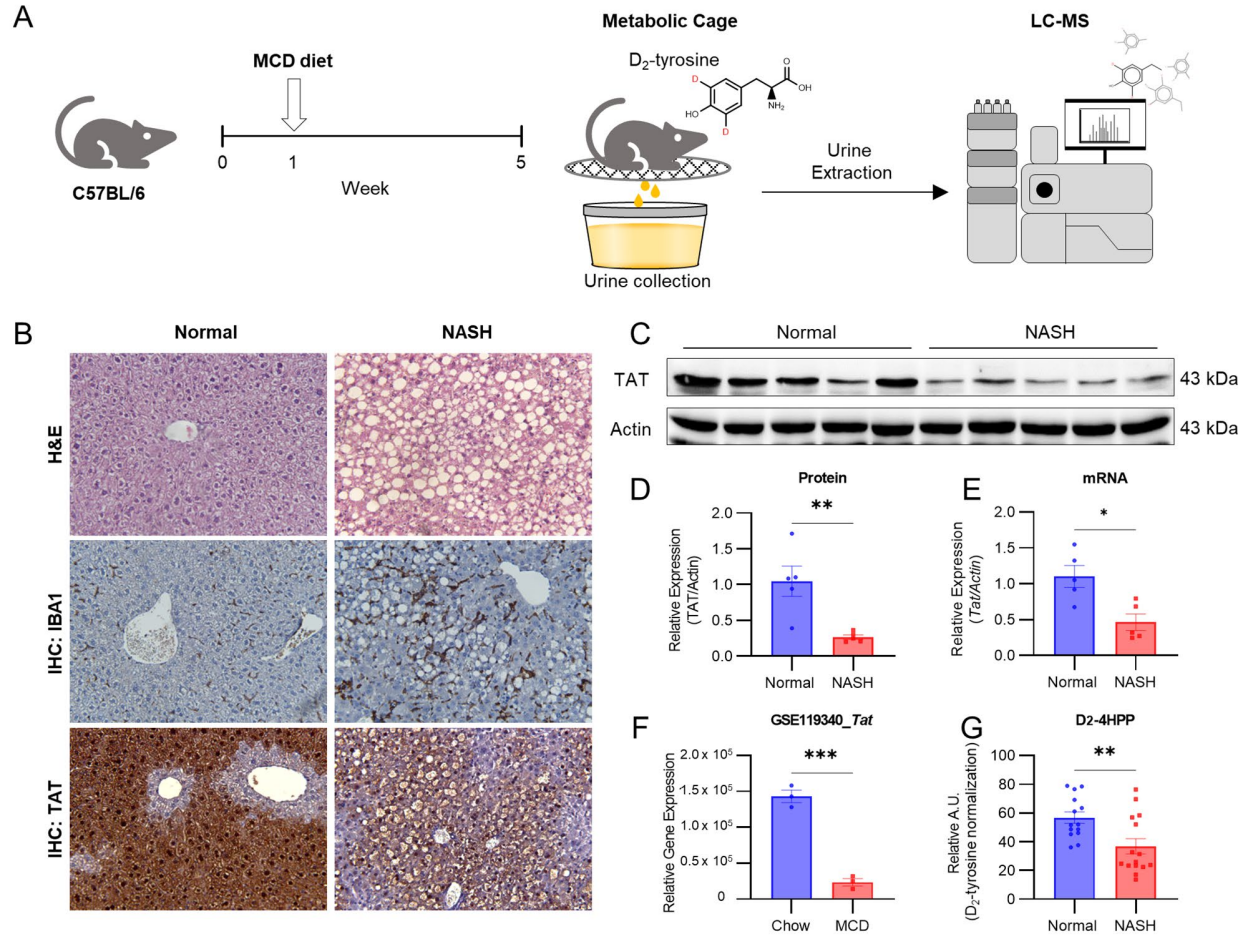
TAT expression in NASH mouse liver tissue. (**A**) Overall scheme of a noninvasive chemical biopsy for the diagnosis of NASH in an MCD-induced mouse NASH model. At week 5 of the MCD diet, D_2_-tyrosine was administered orally and urine was collected from metabolic cages. D_2_-tyrosine and D_2_-4HPP were detected by LC-MS. (**B**) H&E (top), IBA1 (IHC, middle) and TAT (IHC, bottom) images of liver tissues in the MCD diet induced NASH mouse model. The images were taken from livers obtained from mouse sacrificed at 5 weeks after the start of MCD diet. 20x magnification for microscopic images. (**C-E**) Confirmation of TAT expression in normal and NASH liver tissues by qPCR and western blot. Images of the amount of TAT protein (**C**) and quantification of protein (**D**) and mRNA levels (**E**). TAT levels were normalized to actin levels. (**F**) mRNA levels of *Tat* in the MCD mouse model from GSE119340. (**G**) Urinary D_2_-4HPP levels normalized to D_2_-tyrosine levels in normal and NASH mouse models. Statistical analysis was performed by unpaired Student’s *t*-tests. ns. not significant, * *p* < 0.05, ** *p* < 0.01, *** *p* < 0.001, **** *p* < 0.0001

To determine the utility of our chemical biopsy approach in the context of actual NASH disease, the MCD diet of the mouse NASH model was employed (Fig. 3A) (Kucera 2014). Following the well-established protocol, increased ALT, AST levels (Figures S3A, B) and accumulation of lipids in liver cells, as measured in the liver extracts by NMR (Figure S3C), were observed, indicating successful induction of NASH in the mouse model. Consistently, H&E staining exhibited macrophagic steatosis and ballooning degeneration of hepatocytes in the histological morphology (Fig. 3B, top). In addition, IHC staining for IBA1 identified scattered inflammation, characteristic of NASH (Fig. 3B, middle). Most importantly, decreased TAT IHC staining was observed in the liver tissue of the NASH group (Fig. 3B, bottom), which was also confirmed by western blot and mRNA expression of TAT in the liver tissue extracts (Fig. 3C-E). These results were consistent with RNA-seq data from a NASH mouse model generated with the same MCD strategy as our method (GSE119340, Fig. 3F). Confirming that the MCD NASH mouse model was successfully established. Following the administration of D_2_-tyrosine to the mice, urine samples were collected and analyzed to measure the levels of D_2_-4HPP produced by liver TAT using LC-MS (Fig. 3A). The liver TAT activity, which can be estimated by the product (D_2_-4HPP) normalized by the substrate (D_2_-tyrosine), was significantly lower (~ by 36%, *p* = 0.0054) in the NASH group mice (Fig. 3G). These results confirm the feasibility of diagnosing NASH using our noninvasive chemical biopsy.

### Downstream metabolism can be exploited for improved selectivity of the chemical biopsy

**Fig. 4:**
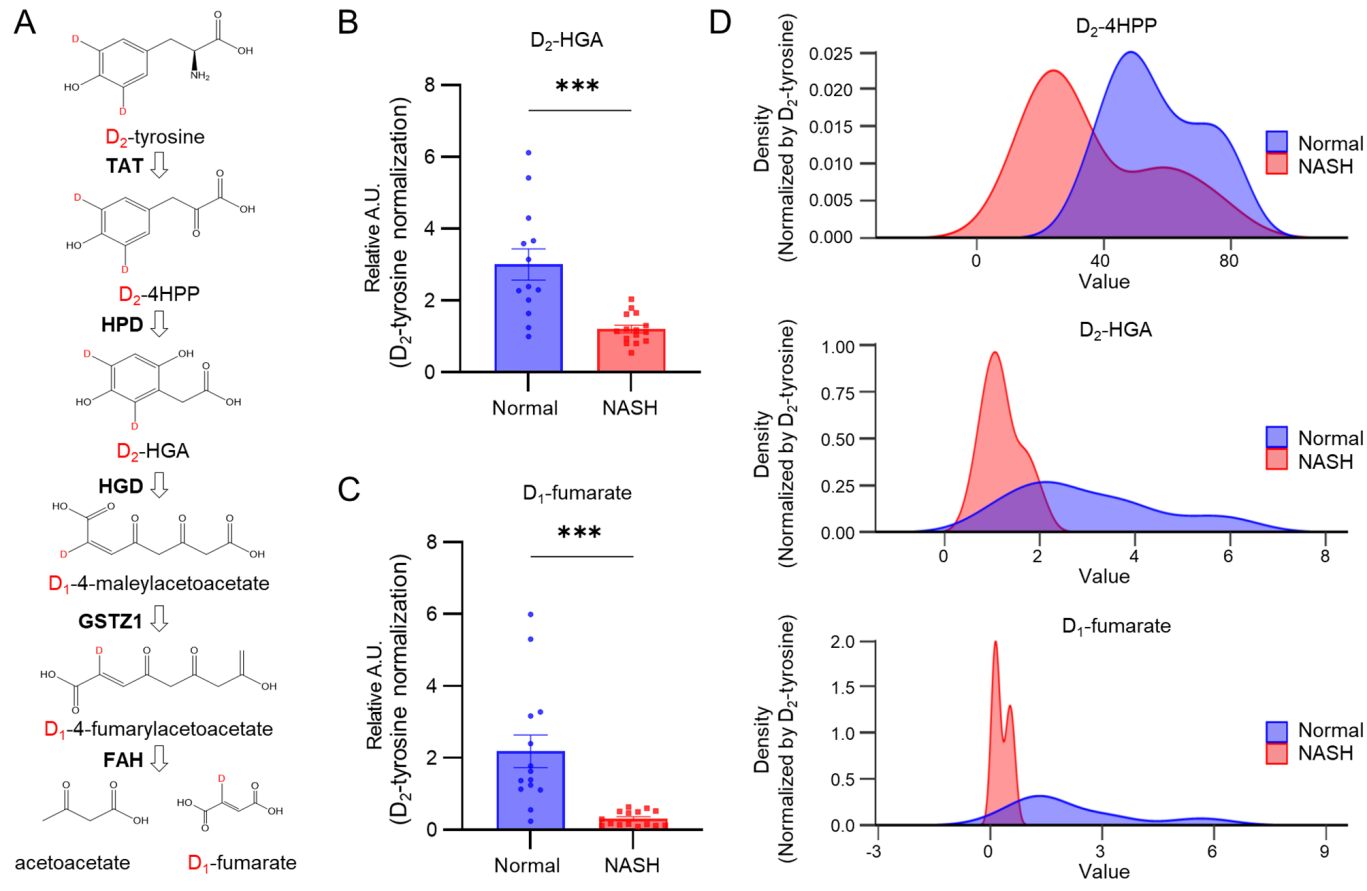
The tyrosine degradation pathway can be used to diagnose NASH. (**A**) Scheme of the D_2_-tyrosine degradation pathway. (**B-D**) Comparison of urinary D_2_-homogentisate (**B**) and D_1_-fumarate (**C**) levels in normal and NASH mice measured by chemical biopsy after administration of D_2_-tyrosine. Urine metabolites, including deuterium, were normalized to D_2_-tyrosine levels. Data were collected from biologically independent mouse urine samples. (**D**) Density plots of metabolites derived from D_2_-tyrosine. From top to bottom: plots of D_2_-4HPP, D_2_-HGA, and D_1_-fumarate. Blue: normal, Red: NASH. Statistical analysis was performed by unpaired Student’s *t*-tests. ns. not significant, * *p* < 0.05, ** *p* < 0.01, *** *p* < 0.001, **** *p* < 0.0001

**Fig. 5:**
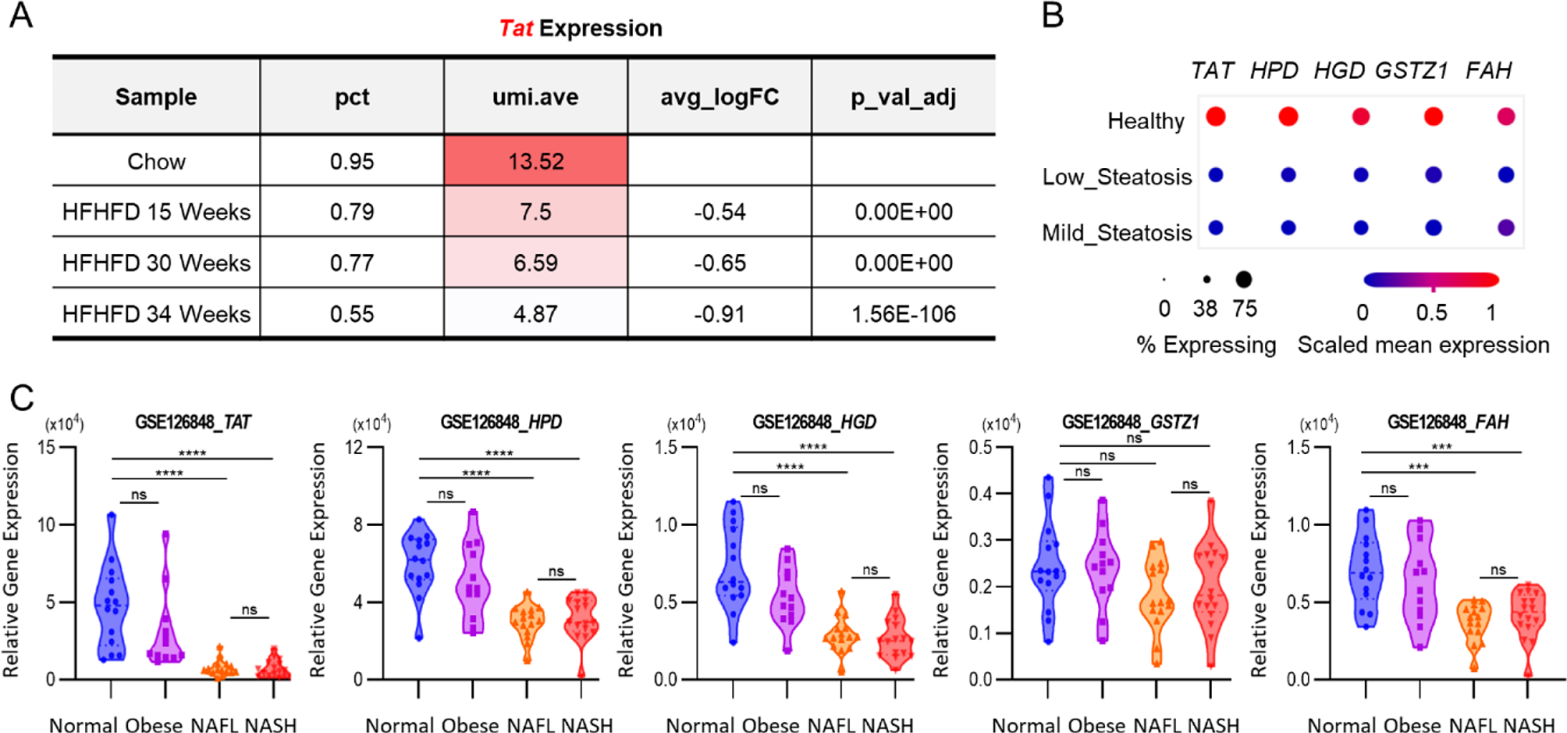
The tyrosine degradation pathway can be used to determine the extent of steatosis. (**A**) Identified changes in scRNA expression of *Tat* gene upon progression of HFHFD diet mouse. Represents NAFL at 15 weeks and NASH at 30 weeks. (**B**) Identified changes in scRNA expression of tyrosine degradation pathway genes upon progression of steatosis. (**C**) GEO data (GSE126848) showing expression of tyrosine pathway enzymes by liver status. Statistical analysis was performed by unpaired Student’s *t*-tests. ns. not significant, * *p* < 0.05, ** *p* < 0.01, *** *p* < 0.001, **** *p* < 0.0001. Data in panel (**B**) was acquired from Single Cell Portal

While the observed decrease in TAT activity is significant in NASH, the modest effect size raises concerns regarding the potential for misdiagnosis. To enhance diagnostic accuracy, we explored the utility of metabolites further downstream in the D_2_-tyrosine metabolic pathway (Fig. 4A). Employing the same strategy developed for D_2_-4HPP, we detected D_2_-homogentisate (HGA), a product of 4HPP metabolism by 4-hydroxyphenylpyruvate dioxygenase (HPD), in urine (Fig. 4B). Similarly, D_1_-fumarate, generated by fumarylacetoacetate hydrolase (FAH) at the terminal stage of the tyrosine degradation pathway, was also detected (Fig. 4C). Upon separate analysis, both D_2_-HGA and D_1_-fumarate levels were markedly reduced in the NASH group compared to control (~ by 60%, ~ by 86%, *p* = 0.00133, *p* = 0.00120), mirroring the pattern observed with D_2_-4HPP (Figs. 3G, 4B-C). This trend indicates that, moving further downstream in the tyrosine degradation pathway, the difference in deuterium-labeled metabolites between groups becomes more pronounced. Additionally, density plot comparisons across groups showed progressively less overlap for metabolites further downstream in the pathway, improving group distinguishability (Fig. 4D). In addition, subsequent gene expression analysis on the tyrosine degradation pathway, utilizing GEO datasets from the same dietary NASH mouse model, revealed significant downregulation of associated genes, including HPD and FAH, from the same dietary NASH mouse model (Figure S4A). The trend was recapitulated in human NASH, with advanced NASH patients showing greater differences than those with milder forms of the disease (Figure S4B). Similarly, when the data was reanalyzed using the High Fat High Fructose diet (HFHFD) model (Su et al. 2021), which represents NAFL (Nonalcoholic fatty liver) at 15 weeks and NASH at 30 weeks, a significant decrease in *Tat* levels in hepatocytes was observed over time (Fig. 5A, S4C). In addition, a significant decrease in gene expression in the tyrosine degradation pathway was observed, when we analyzed the time dependence in a dataset of steatosis progression (Fig. 5B) (Fabre et al. 2023). Importantly, genes associated with the tyrosine degradation pathway were not decreased in obesity compared to normal but were decreased in steatosis and NASH (Fig. 5C). Taken together, these results confirm that our chemical biopsy approach reflects the level of tyrosine degradation pathway in liver tissue during the development of NASH. They further advocate for the use of D_2_-tyrosine and its metabolites D_2_-4HPP, D_2_-HGA, and D_1_-fumarate, as potential noninvasive diagnostic tools for NASH diagnosis.

## Discussion

This study introduces a new noninvasive chemical biopsy technique for the diagnosis of NASH. Current noninvasive diagnostic methods of NASH can be broadly divided into two categories: the first being imaging techniques, such as ultrasound or magnetic resonance techniques, that measure the physical state of the liver; the second being liquid biopsy methods that measure biomarkers in the blood (Castera et al. 2019), (Zhou et al. 2019). In principle, liquid biopsy can involve urine or blood, but there are no pathology-relevant urine biomarkers for NASH. The imaging methods, although detecting liver status directly, still have drawbacks. For example, FibroScan^®^, a specialized form of ultrasonography measuring liver stiffness and approved for liver fibrosis, may not be sensitive to early inflammatory stages of NASH, where interventions will be most helpful. In addition, the presence of large visceral fat can compromise the accuracy of the approach, which is problematic given the established relationship between obesity and NASH (Francque et al. 2021), (Siddiqui et al. 2018). The approach also requires expertise of medical professionals, although less so than magnetic resonance imaging, and the results are prone to subjective experience. In comparison, liquid biopsy methods are more objective and has good inter-laboratory reproducibility, and are much cheaper. The major drawback of liquid biopsy, on the other hand, is the specificity to NASH, as the markers may be elevated in unrelated conditions. For example, AST/ALT, commonly measured for liver test, are not specific for inflammation or NASH (Kim et al. 2008), (Kew 2000), and may be increased even in obesity (Ruhl and Everhart 2003). Other more specialized biomarkers such as CK18, CXCL10, and FGF21 can provide information related to cell death, inflammation, fat metabolism, etc., but they are not specific to the liver pathology (Fedchuk et al. 2014). Recently, efforts have been made to improve the accuracy of NASH diagnosis by developing indices and profiles that combine blood-based markers with liver stiffness (Srivastava et al. 2019), (Newsome et al. 2020), (Harrison et al. 2020), but controversy still exists as to whether they represent a specific response of hepatocytes to NASH disease.

Nevertheless, the field of liquid biopsy analysis has significant potential and is developing rapidly (Felden et al. 2020), (Nikanjam et al. 2022), (Zhou et al. 2022). In this regard, our approach employing TAT and other enzymes in the tyrosine degradation pathways with urine samples may feature several benefits. D_2_-tyrosine intake and urine collection can be performed by patients without the physical assistance of a healthcare professional. This provides convenience in sampling, as even blood markers may require hospital visits. In addition, the approach can be integrated directly into the routine LC-MS procedures used in many hospitals, and the results can be objectively determined without experienced medical personnel. Another aspect of our chemical biopsy is the use of one tracer, D_2_-tyrosine, to measure the decrease in three metabolites (D_2_-4HPP, D_2_-HGA, D_1_-fumarate) along the single tyrosine degradation pathway, providing confidence in the overall measurement. This measurement can be easily implemented in a single LC-MS run with a multiple reaction monitoring procedure without the need to measure the metabolites separately. Compared to many other urine metabolic markers that are simply observational and not related to actual target organ pathology, the tyrosine metabolism is highly specific to liver and exhibited correlation with NASH development, as we showed above. Although 4HPP and HGA have not been measured, these trends are consistent with those previously reported for tyrosine and fumarate in NASH patients’ serum (Table S5). Except for sophisticated imaging techniques, liver specific changes are monitored mostly with tissue biopsy, and there are few liver specific liquid biopsy biomarkers. Given this, it may be natural that liver tissue biopsy is the gold standard for NASH diagnosis. Our design of chemical biopsy procedure and careful selection of markers made it possible to monitor liver specific metabolic changes with urine, even without the need to blood drawing. In that regard, it is notable that tyrosine degradation pathway did not change due to obesity but significantly decreased with the progression of fatty liver disease (Fig. 5B, C). This may represent additional specificity benefit compared to commonly used liver markers such as AST/ALT. Still, there is a possibility that TAT decrease is also observed in hepatocellular carcinoma. As it has recently been suggested that tyrosine degradation dysfunction is an early event that contributes to the transformation and development of HCC (Hepatocellular carcinoma)  (Tong et al. 2021), it is possible that early dysfunctions accumulate and progress in a time-dependent manner from steatosis to NASH to HCC.

Besides presenting practical merits in NASH diagnosis, our study provides a possibility that reduced TAT activity in NASH patients may be important in the pathology of NASH. First, it has been reported that excessive nutrition and fatty acid supply not only promote TAT degradation in an ATP- and ubiquitin-dependent manner (Gross-Mesilaty et al. 1997), but also induces oxidative stress, leading to the generation of abnormal tyrosine isomers (meta- and ortho-) (Pessayre and Fromenty 2005). These tyrosine isomers further promote abnormal protein degradation, inhibition of cell proliferation, and induction of apoptosis (Ipson and Fisher 2016), (Dunlop et al. 2008). The enhanced protein degradation might be associated with the high levels of amino acids observed in the blood in NASH patients. Alternatively, accumulated tyrosine in the liver can be converted to false neurotransmitters (tyramine, octopamine, L-dopa) (Channer et al. 2023) that can be converted to dopamine by liver cytochrome P450 2D6 (Hiroi et al. 1998). This dopamine then acts on macrophages to increase the production of the chemokines CXCL9 and CXCL10, which recruit CD8^+^ T-cells to enhance the immune response (Channer et al. 2023). Second, the reduction in tyrosine degradation pathway will lead to the decrease in the resulting metabolites such as 4-HPP and fumarate that have been shown to inhibit inflammation and T-cell activation. For example, 4-HPP was shown to inhibit NLRP3 inflammasome activation during the LPS-induced septic response in a mouse model (Wei et al. 2022). For fumarate, its succination of GAPDH in immune cells was suggested to underlie the immunomodulatory activity of dimethylfumarate, an FDA-approved drug to treat multiple sclerosis, an auto-immune inflammatory disease (Kornberg et al. 2018), (Cheng et al. 2023). In addition, depletion of cancer cell-derived fumarate in the tumor microenvironment may be a way to stimulate inflammatory responses and improve the efficacy of immunotherapies such as chimeric antigen receptor (CAR)-T cell therapy (Cheng et al. 2023). Third, in glioma, increased protein levels of the tyrosine degradation pathway (HPD, HGD, and FAH) was correlated with higher PD-L1 expression, which promoted immune evasion by suppressing T-cell activity (Wang et al. 2023). In NASH cases, this would translate into lower PD-L1 (CD274) expression, which, indeed, was found upon the analysis of a GEO dataset (Figure S4D). This suggests that lower tyrosine degradation may be associated with lower immune checkpoint activity, leading to the inflammatory response. Interestingly, some liver cancer stem cells (CD13^+^, candidate liver cancer stem cell marker) rely on aerobic metabolism of tyrosine, rather than glucose, as an energy source (Sun et al. 2020). This suggests that the non-metabolized tyrosine during the progression of NASH might help cancer stem cells to grow and develop liver cancer. Taken together, reduced tyrosine degradation pathway may not only be a biomarker but also be closely associated with pathogenesis of NASH.

With these considerations, our study is limited by the animal NASH models (our MCD diet and analysis on a previous HFHFD model) and the number of samples. Although we provided consistent results of TAT expression from human cohort data, further human studies are warranted to fully evaluate the applicability of the proposed chemical biopsy approach to human NASH diagnosis. In addition, further mechanistic study focusing on the tyrosine degradation pathway is expected to contribute to an increased understanding of the pathogenesis of NASH and the development of new therapeutic strategies.

## Conclusions

Our study introduces TAT as a novel NASH marker and demonstrates that noninvasive chemical biopsy targeting the liver-specific tyrosine pathway holds promise for the diagnosis and management of NASH. The reduced tyrosine degradation pathway might be involved in the pathology of NASH development.

## Electronic supplementary material

Below is the link to the electronic supplementary material.

Supplementary Material 1

Supplementary Material 2

## Data Availability

No datasets were generated or analysed during the current study.

## Abbreviations

4HPP: 4-hydroxyphenylpyruvate
AA: Amino acid
AAA: Aromatic amino acid
ACN: Acetonitrile
ALT: Alanine aminotransferase
AST: Aspartate aminotransferase
BCAA: Branched chain amino acid
CT: Computed tomography
FAH: Fumarylacetoacetate hydrolase
GEO: Gene expression omnibus
GSTZ1: Glutathione s-transferase zeta 1
HGA: Homogentisate
HGD: Homogentisate oxidase
HPA: Human protein atlas
HPD: 4-hydroxyphenylpyruvate dioxygenase
IACUC: Institutional animal care and use committees
IHC: Immunohistochemistry
KEGG: Kyoto encyclopedia of genes and genomes
LC-MS: Liquid chromatography-mass spectrometry
MCD: Methionine choline deficient
MRI: Magnetic resonance imaging
MRM: Multiple reaction monitoring
MASLD: Metabolic dysfunction-associated steatotic liver disease
MASH: Metabolic dysfunction-associated steatohepatitis
NAFL: Nonalcoholic fatty liver
NASH: Nonalcoholic steatohepatitis
NIT: Noninvasive testing
TAT: Tyrosine aminotransferase
