# Supplementary material for: TAT as a new marker and its use for noninvasive chemical biopsy in NASH diagnosis
Source: Mol Med. 2024 Nov 26;30:232. doi: 10.1186/s10020-024-00992-8 (PMC11590374; doi:10.1186/s10020-024-00992-8)
Supplement: Supplementary file 2 — Supplementary Material 2 [file 10020_2024_992_MOESM2_ESM.docx]

**TAT as a new marker and its use for noninvasive chemical biopsy in NASH diagnosis**

Sihyang Jo^1^, Jin-Mo Kim^1^, Minshu Li^1^, Han Sun Kim^1, 2^, Yong Jin An ^*,1^ and Sunghyouk Park^*,1^

^1^ Natural Products Research Institute, College of Pharmacy, Seoul National University, 1 Gwanak-ro, Gwanak-gu, Seoul 08826, Korea

^2^ Department of Biochemistry, College of Medicine, Dongguk University, Gyeongju, 38066, Republic of Korea

^*^Correspondence:

**Sunghyouk Park**

Natural Products Research Institute, College of Pharmacy, Seoul National University, Gwanak-Ro 1, Gwanak-gu, Seoul 08826, Republic of Korea, Tel: +82-2-880-7831; Fax: +82-2-880-7831; E-mail: psh@snu.ac.kr

**Yong Jin An**

Natural Products Research Institute, College of Pharmacy, Seoul National University, Gwanak-Ro 1, Gwanak-gu, Seoul 08826, Republic of Korea, Tel: +82-2-880-7834; Fax: +82-2-880-7834; E-mail: biochem.yong@gmail.com

**Supplementary Tables**

**Supplementary Table 1. GEO Datasets for marker detection strategies**

Data for this table was provided the file separately as an Excel.

**Supplementary Table 2. RNA tissue specificity of the top 10 genes with significant changes in the Volcanoplot.**

| **Gene** | **RNA tissue specificity** | **Tissue** |
| --- | --- | --- |
| *ALDH3A1* | Tissue enhanced | esophagus, salivary gland, stomach |
| *ALDH6A1* | Group enriched | kidney, liver |
| *ASS1* | Group enriched | kidney, liver |
| *BCAT1* | Tissue enhanced | pancreas |
| *CPS1* | Tissue enriched | liver |
| *EZH2* | Tissue enhanced | bone marrow, lymphoid tissue, testis |
| *GNMT* | Group enriched | liver, pancreas |
| *HMGCS1* | Tissue enhanced | liver |
| *TAT* | Tissue enriched | liver |
| *P4HA1* | Low tissue specificity | - |

RNA tissue specificity of the top 10 genes with significant changes in the Volcanoplot. Tissue specificity information is taken from HPA. RNA specificity categories are based on mRNA expression levels in the consensus dataset calculated from RNA expression levels in HPA and GTEx samples. Categories include tissue-enriched, group-enriched, tissue-enriched, low tissue specificity, and undetectable.

**Supplementary Table 3. Expression of *TAT* in human normal tissues.**

| **Tissue** | **nTPM** | **Tissue** | **nTPM** |
| --- | --- | --- | --- |
| Adipose tissue | 0 | Midbrain | 0 |
| Adrenal gland | 0 | Ovary | 0 |
| Amygdala | 0 | Pancreas | 0 |
| Basal ganglia | 0 | Pituitary gland | 0 |
| Breast | 14.7 | Prostate | 0 |
| Cerebellum | 0 | Retina | 0 |
| Cerebral cortex | 0 | Salivary gland | 0 |
| Cervix | 0 | Skeletal muscle | 0 |
| Colon | 0 | Skin | 0 |
| Endometrium | 0 | Small intestine | 0 |
| Esophagus | 0 | Spinal cord | 0 |
| Fallopian tube | 0 | Spleen | 0 |
| Heart muscle | 0 | Stomach | 0 |
| Hippocampal formation | 0 | Testis | 0 |
| Hypothalamus | 0 | Thyroid gland | 0 |
| Kidney | 0 | Urinary bladder | 0 |
| Liver | 838.8 | Vagina | 0 |
| Lung | 0 |  |  |

The RNA expression of the normal tissues was normalized using the internal normalization pipeline. Expression (nTPM) levels for 35 tissue types, created by combining the HPA and GTEx transcriptomics datasets

**Supplementary Table 4. Liver tissue enriched gene list and metabolic reaction among genes altered in NASH.**

| **Gene** | **RNA tissue specificity** | **Tissue** | **Description** | **Reaction** |
| --- | --- | --- | --- | --- |
| *CPS1* | Tissue enriched | liver | carbamoyl-phosphate synthase 1 | 2 ATP + bicarbonate + NH4^+^ = 2 ADP + carbamoyl phosphate + 2 H^+^ + phosphate |
| *TAT* | Tissue enriched | liver | tyrosine aminotransferase | L-tyrosine + 2-oxoglutarate = 4-hydroxyphenylpyruvate + L-glutamate |

List of liver tissue enriched genes and metabolic responses among genes altered in NASH. For the enzyme reactions, they were obtained from Rhea (rhea-db.org).

**Supplementary Table 5. Literature summary of metabolites related to tyrosine degradation in metabolomics data from previous studies using NASH patients.**

| Sample | Body fluids | Metabolite / Metabolism | Tendency | Statistical Value | Author | Journal |
| --- | --- | --- | --- | --- | --- | --- |
| human | plasma | tyrosine | increase | 0.0001 | Kalhan SC, et al. | (1) |
| human | liver | tyrosine | increase | ≤ 0.05 | Lake AD, et al. | (2) |
| human | plasma | tyrosine | increase | <.001 | Jin R, et al. | (3) |
| human | serum | tyrosine | increase | 0.001 | Sookoian S, et al. | (4) |
| human | serum | fumaric acid or  maleic acid | decrease | 0.04 |  |  |
| human | urine | tyrosine | increase | 0.011 | Dong S, et al. | (5) |
| human | plasma | tyrosine | increase | < 0.001 | Yamakado M, et al. | (6) |
| human | plasma | tyrosine | increase | 0.005 | Gaggini M, et al. | (7) |
| human | serum | tyrosine | increase | < 0.001 | Hasegawa T, et al. | (8) |
| human | serum | tyrosine | increase | < 0.001 | Mello VD, et al. | (9) |

**Supplementary Figure legends**

**Supplementary Figure 1. Strategy for finding biomarkers in NASH.** (A) Metabolite set enrichment analysis based on high amino acid in NASH. (B) Volcano plot showing differentially expressed genes between Normal liver with NASH liver. The X-axis shows the fold change, while the Y-axis shows the −log_10_ (*p*-value). Genes with a Log_2_ fold change >0.5 in NASH were color coded: genes that decreased were shown in blue, genes that increased were shown in red, and the top 10 genes with the largest fold change were shown with nametags. (C-D) Single‑cell RNA‑seq data for expression of *TAT* in liver cell type. (C) Annotation of the cell types within the UMAP based on transcriptome (left). mRNA expression levels of *TAT* (right). Hepatocytes are indicated with red circles. (D) Prediction of RNA biomarkers of hepatocyte type clusters generated from Figure 1C. Data in panels (C) was acquired from human liver dataset in Single Cell Portal (https://singlecell.broadinstitute.org/single_cell). Data in panels (D) was acquired from human liver dataset in Azimuth (https://app.azimuth.hubmapconsortium.org/app/ human-liver).

**Supplementary Figure 2. Confirmation of TAT expression in primary hepatocytes using western blot.** For protein analysis, a 12% SDS gel was used. After lysing the cells as described in the Methods section, protein concentration was determined using the BCA method, and 20 μg of each sample was loaded into the wells. When primary hepatocytes were exposed alongside control cell lines HepG2, Hep3B (Hepatocellular carcinoma), and SNU423 (Pleomorphic hepatocellular carcinoma; Grade III/IV), the actin band in the primary hepatocytes was not clearly visible (far left). Therefore, the membrane was cut, and only the primary hepatocyte samples were re-exposed separately (center).

**Supplementary Figure 3. MCD-fed mice exhibit physiologic features of NASH.** (A and B) Serum AST and ALT levels after 5 weeks of MCD feeding in a mouse NASH model. Serum AST (A) and ALT (B) levels were measured by biochemical blood analyzer. AST, aspartate aminotransferase; ALT, alanine aminotransferase. (C) Determining lipid levels in normal and NASH mouse liver tissue using NMR. Statistical analysis was performed by unpaired Student’s *t*-tests. ns. not significant, * *p* < 0.05, ** *p* < 0.01, *** *p* < 0.001, **** *p* < 0.0001.

**Supplementary Figure 4. Confirm expression in NASH of genes in the tyrosine degradation pathway.** (A) mRNA levels of tyrosine degradation pathway enzymes in liver tissue from normal and NASH mouse models. (B) mRNA levels of tyrosine degradation pathway enzymes with progression of NASH patients. (C) Identified changes in scRNA expression of *Tat* gene upon progression of HFHFD diet mouse NASH. (D) GEO data showed that the expression of PD-L1 (CD274) in NASH. Statistical analysis was performed by unpaired Student’s *t*-tests. ns. not significant, * *p* < 0.05, ** *p* < 0.01, *** *p* < 0.001, **** *p* < 0.0001.

**Supplementary Figures**

Supplementary Figure 1.

**
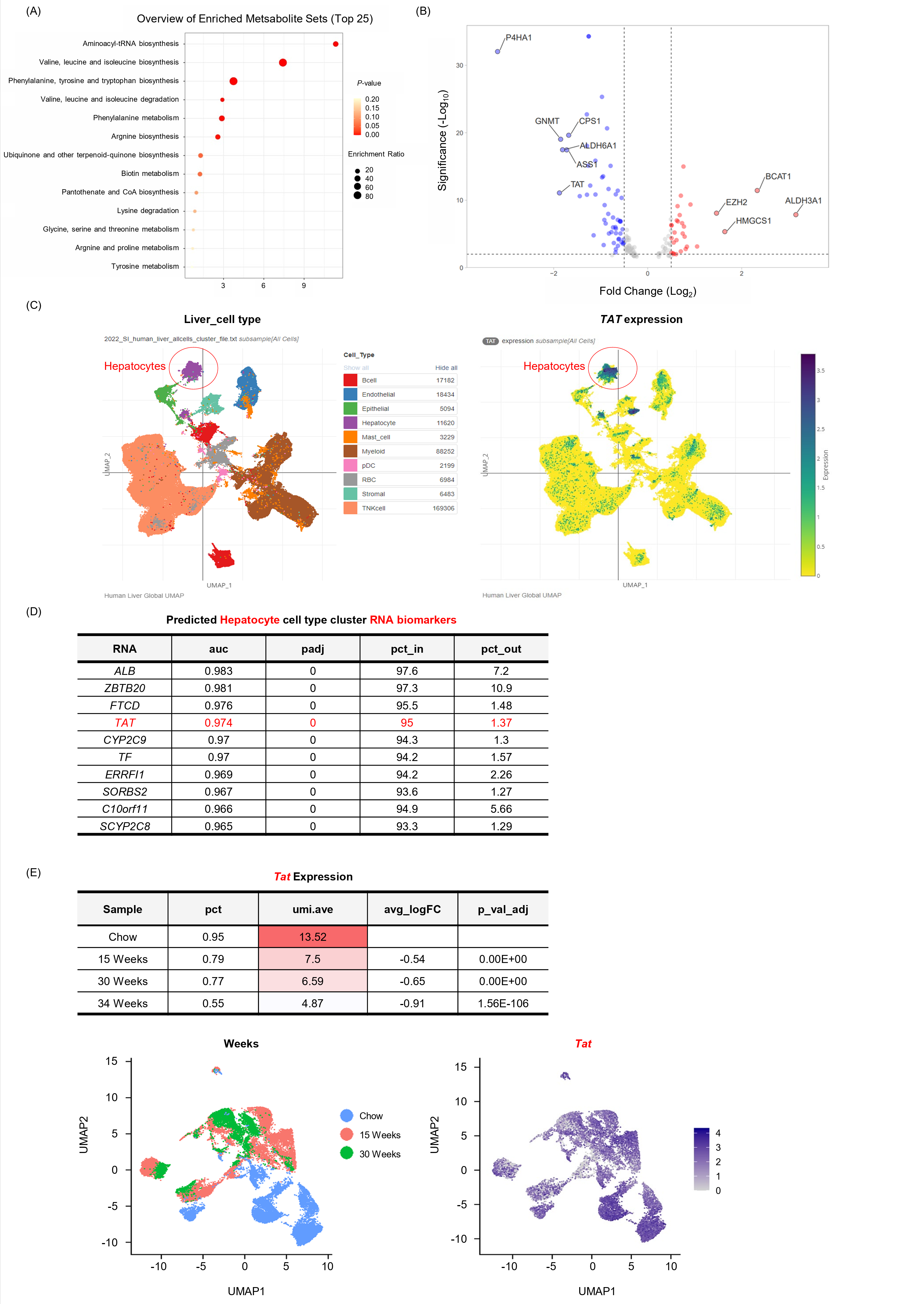
**

Supplementary Figure 2.


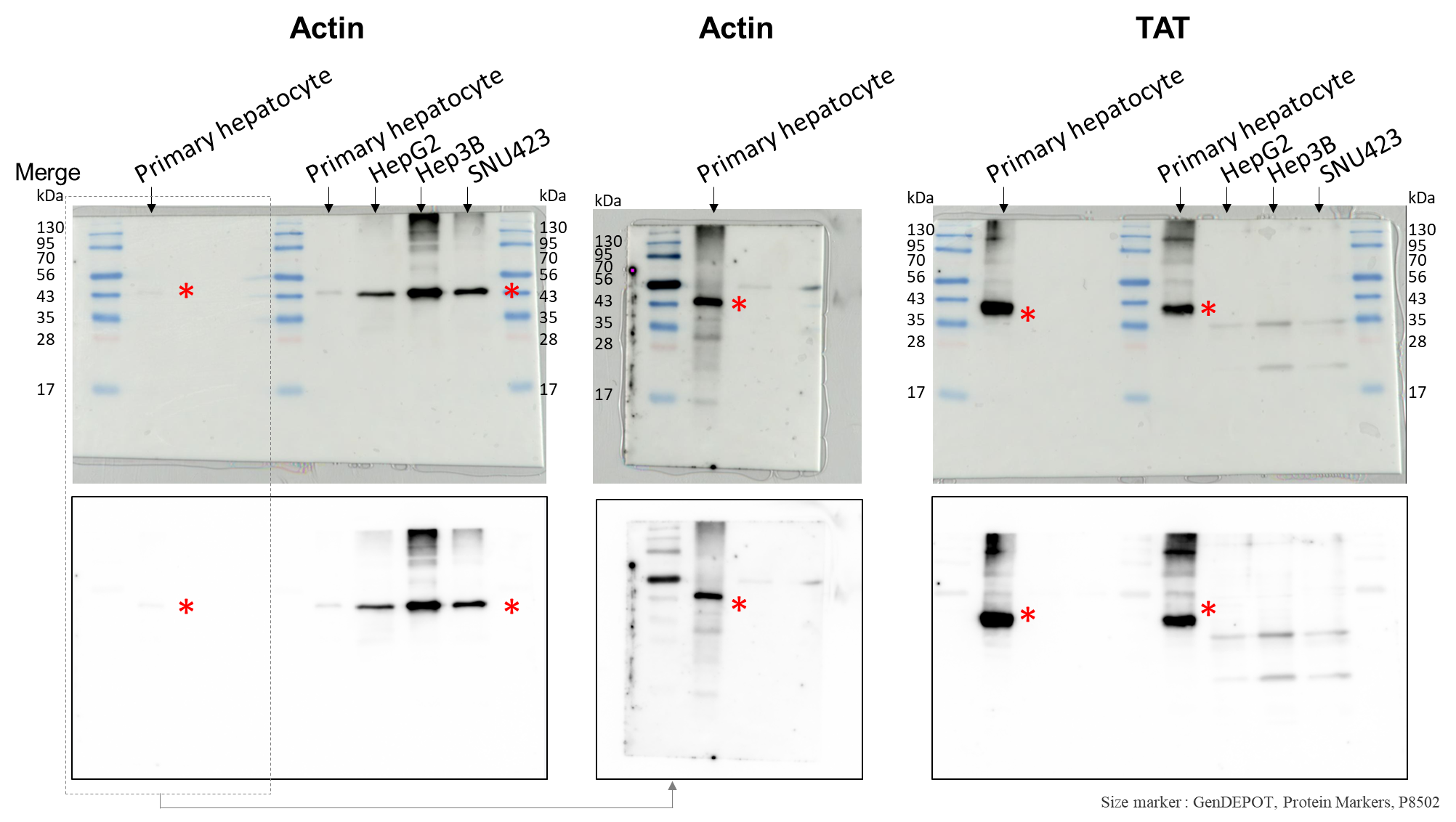


Supplementary Figure 3.

**
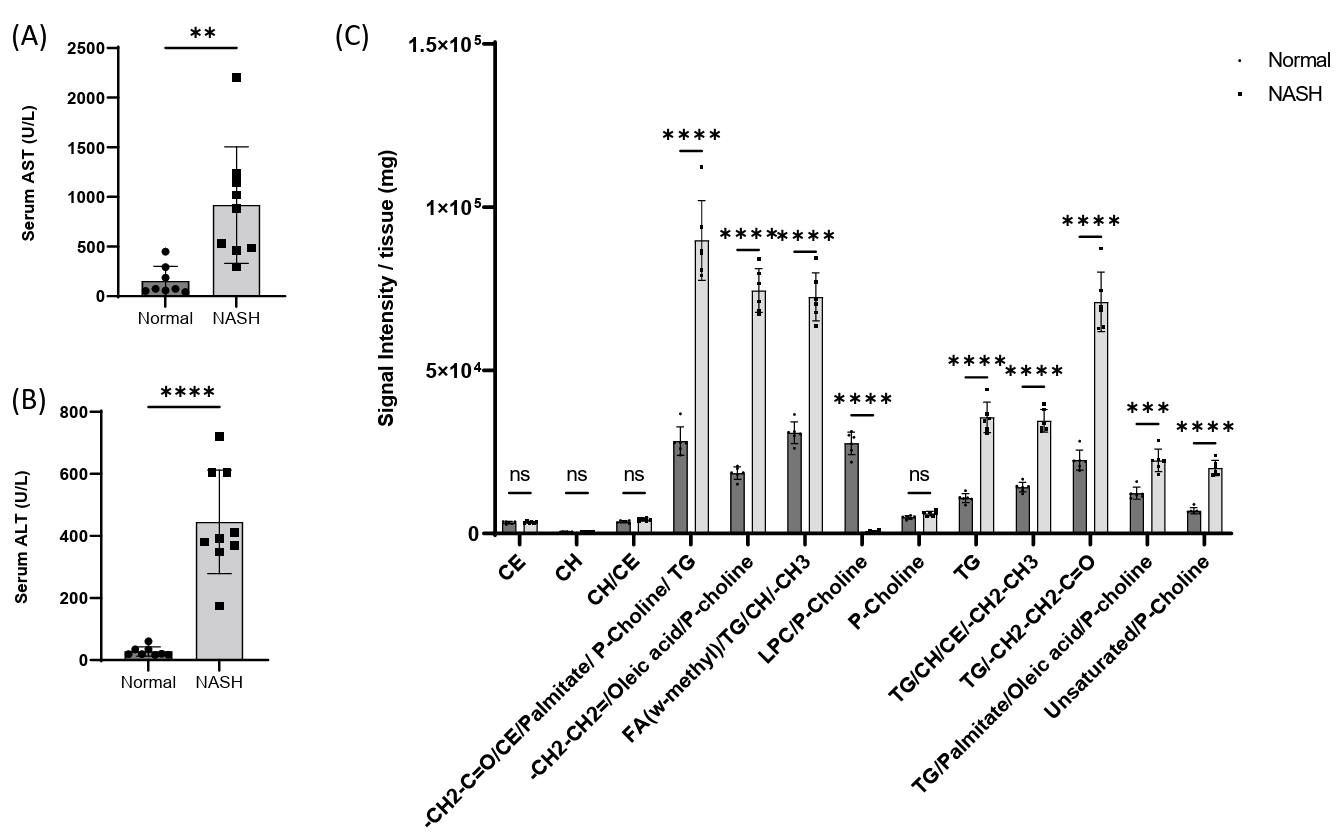
**

Supplementary Figure 4.

**
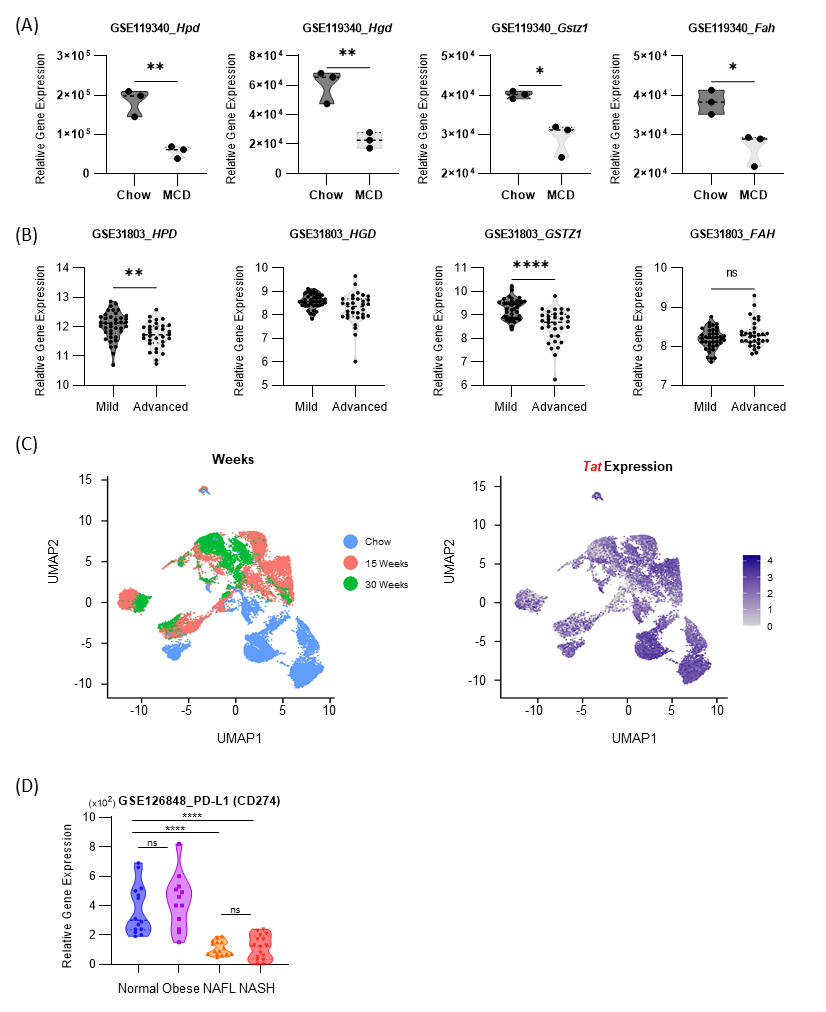
**

**References**

1. Kalhan SC*, et al.* (2011) Plasma metabolomic profile in nonalcoholic fatty liver disease. *Metabolism* **60:** 404-413.

2. Lake AD*, et al.* (2015) Branched chain amino acid metabolism profiles in progressive human nonalcoholic fatty liver disease. *Amino Acids* **47:** 603-615.

3. Jin R*, et al.* (2016) Amino Acid Metabolism is Altered in Adolescents with Nonalcoholic Fatty Liver Disease-An Untargeted, High Resolution Metabolomics Study. *The Journal of Pediatrics* **172:** 14-19 e15.

4. Sookoian S*, et al.* (2016) Serum aminotransferases in nonalcoholic fatty liver disease are a signature of liver metabolic perturbations at the amino acid and Krebs cycle level. *American Journal of Clinical Nutrition* **103:** 422-434.

5. Dong S*, et al.* (2017) Urinary metabolomics analysis identifies key biomarkers of different stages of nonalcoholic fatty liver disease. *World J Gastroenterol* **23:** 2771-2784.

6. Yamakado M*, et al.* (2017) Plasma amino acid profile associated with fatty liver disease and co-occurrence of metabolic risk factors. *Scientific Reports* **7:** 14485.

7. Gaggini M*, et al.* (2018) Altered amino acid concentrations in NAFLD: Impact of obesity and insulin resistance. *Hepatology* **67:** 145-158.

8. Hasegawa T*, et al.* (2020) Changed Amino Acids in NAFLD and Liver Fibrosis: A Large Cross-Sectional Study without Influence of Insulin Resistance. *Nutrients* **12:** 1450.

9. Mello VD*, et al.* (2021) Serum aromatic and branched‐chain amino acids associated with NASH demonstrate divergent associations with serum lipids. *Liver International* **41:** 754-763.
